# Between the ambulance and academia: rethinking identity and competence in paramedics with reduced clinical exposure

**DOI:** 10.29045/14784726.2026.3.10.4.3

**Published:** 2026-03-01

**Authors:** Caitlin Wilson, James Wilkinson, Ellie Hilton, Larissa Prothero, Sharon Seddon, Laura Blair, David Fitzpatrick

**Affiliations:** Yorkshire Ambulance Service NHS Trust; University of Hertfordshire ORCID iD: https://orcid.org/0000-0002-9854-4289; East of England Ambulance Service NHS Trust; University of Hertfordshire ORCID iD: https://orcid.org/0009-0001-9959-4632; London Ambulance Service NHS Trust; University of Hertfordshire ORCID iD: https://orcid.org/0009-0000-0793-7443; East of England Ambulance Service NHS Trust ORCID iD: https://orcid.org/0000-0002-5440-8429; University of Cumbria; North West Ambulance Service NHS Trust ORCID iD: https://orcid.org/0000-0003-1661-646X; North East Ambulance Service NHS Foundation Trust ORCID iD: https://orcid.org/0000-0001-9846-9429; University of Stirling; University of Tasmania ORCID iD: https://orcid.org/0000-0003-0653-8445

The evolution of paramedicine into a diverse and academically grounded profession has expanded career pathways well beyond frontline clinical practice in operational ambulance settings. Paramedics now commonly work as researchers, lecturers, clinical academics and educators, as well as in specialist advisory or project roles, both within and outside ambulance services. These developments reflect both the maturation of the profession and the appeal of portfolio careers that combine operational shifts with roles in higher education, research and leadership. Dual- or multi-role clinicians often bring with them enhanced critical thinking, deeper engagement with evidence and the ability to identify research questions directly arising from frontline clinical problems. By translating practice-based challenges into focused inquiry, clinical academics help generate new knowledge and improve patient care. Their own knowledge and experiences from clinical practice can be shared with student paramedics and other colleagues to facilitate greater learning opportunities, to support research supervision and to create valuable case studies for discussion. Such diversification strengthens the discipline’s academic foundations and supports professional longevity, but it also provokes an enduring question: how can paramedics maintain clinical competence and confidence when practising clinically less than full time?

This editorial initially sought to reflect on the specific experiences of research and academic paramedics, who frequently report challenges in sustaining operational practice. However, through discussion and engagement with wider evidence, it became clear that reduced clinical exposure affects a much broader group. Paramedics in rural settings often encounter fewer patients: a median of seven patients per week, compared to 10 patients for their urban-based counterparts (Pilbery, 2025). Those on specialist secondments or development pathways may experience prolonged periods with limited clinical shifts. Clinicians returning from sickness, injury or parental leave face the well-recognised difficulty of rebuilding confidence and familiarity with evolving practice (Dod & Lansdown, 2025). Even full-time clinicians may experience reduced exposure due to extended hospital handover delays limiting the number of patients they attend.

## Paramedics in academia and research: identity and professional transition

The growth of paramedics in academia represents a major professional shift. Paramedic lecturers and researchers contribute to evidence-based practice, curriculum innovation and disciplinary theory (Naidoo et al., 2024; Sheahan et al., 2025). Yet, this evolution has also triggered questions of identity and belonging, and in some instances, feelings of imposter syndrome.

Munro et al. (2018) coined the term ‘no man’s land of professional identity’ to describe the experience of paramedics transitioning from frontline roles into academia. Moving from an area of familiarity and comfort as a clinician into less-acquainted territory as an academic can result in deep, internal moral conflict. Knights and Clarke (2014) highlight this dilemma as the ‘fragile academic self’, which can be explained as the desire to maintain a core identity as a practitioner, while not entirely resonating as being academic. Those working in this space must attempt to internally bridge the gap between the two identities, fostering a sense of belonging in both and ultimately becoming a ‘pracademic’ (Dickinson et al., 2022). Wilson et al. (2022) illuminated these identity challenges, showing how the fragile academic self is clearly visible, even in the terminology individuals use to introduce themselves – such as choosing between ‘paramedic’, ‘research paramedic’ or ‘clinical academic’ – which reflects an ongoing negotiation between clinical and scholarly identities. This fluidity underscores both the richness and the fragility of professional identity among paramedics working across domains.

Many new academics report uncertainty about their legitimacy in both contexts, compounded by limited research experience and structural barriers (Gratrix et al., 2025; Munro et al., 2016a, 2016b, 2019).

## Clinical exposure, systematic barriers and portfolio practice

A key aspect of this identity crisis is reduced clinical exposure, with Ross et al. (2023) observing that some paramedic academics abandon their clinical practice altogether due to a lack of time and perceived support for clinical activity within academia. Universities lack clear frameworks or expectations regarding clinical currency for paramedic academic staff, resulting in inconsistent approaches and uncertainty about what constitutes adequate maintenance of competence. Conversely, healthcare organisations often have a predominantly clinical focus, so gaining experience by undertaking research, applying for grant funding or writing for publication can be harder for frontline paramedics. For those pursuing these paths, academia may offer better opportunities.

Where clinical exposure and practice are limited, there will inevitably be knowledge and skills decay (Campbell et al., 2019; Yang et al., 2012). Studies have investigated clinicians’ knowledge and skills decay over time, with evidence consistently demonstrating a negative correlation between time out of practice and skill retention; the longer the interval, the greater the decline (Arthur Jr et al., 1998; Caddick et al., 2023; Yang et al., 2012). Variation exists regarding how rapidly deterioration occurs – within as little as three months or longer – but the trend is consistent (Caddick et al., 2023; Campbell et al., 2019; Fisher et al., 2018; Hamilton, 2005; Lee et al., 2011; Mosley & Shaw, 2013; Yang et al., 2012). Evidence linking inadequate resuscitation training to poorer patient outcomes further reinforces that reduced exposure to high-acuity events in particular has genuine implications for competence and care quality (Bobrow et al., 2013; Dane et al., 2000; Moretti et al., 2007). This wider context illustrates that reduced exposure is not a phenomenon confined to paramedics undertaking academic work. It is a systemic issue affecting diverse parts of the workforce, reinforcing the need for thoughtful, co-ordinated approaches to competence maintenance.

Maintaining clinical practice alongside an academic role presents significant barriers: demanding workloads, competing performance metrics and logistical obstacles, such as rostering and access to continued professional development (CPD). Training days, mandatory updates and practical refreshers are often scheduled on fixed cycles, which may not align with part-time clinical contracts. As a result, part-time clinicians may be required to take annual leave or unpaid time to attend essential training or risk missing sessions, which could lead to a requirement for additional retraining. This creates inequitable burdens on those in academic posts and contributes to attrition from clinical practice. In some zero-hour clinical contracts, minimum requirements must be met within set periods to maintain the contract, creating further pressure to work weekends and rest days as overtime on top of normal contracted academic hours, all while trying to uphold a safe work‒life balance.

Such challenges are compounded by the regulatory context: in the UK, frontline ambulance practice is not required for continued registration with the Health and Care Professions Council (HCPC). While this flexibility supports diversification within the profession, it also reduces structural incentives for organisations to protect operational shifts for staff in non-clinical roles. As a result, paramedics who wish to maintain part-time clinical activity must navigate a complex landscape of competing demands and limited support.

Despite such challenges, these individuals embody the profession’s clinical‒academic bridge, linking frontline realities with educational and research innovation (Joyce et al., 2009; Wilson et al., 2022). Importantly, engagement in research can also enhance clinical capability, deepening understanding of evidence, fostering interdisciplinary collaboration and strengthening critical appraisal skills. In this way, portfolio practice may enrich clinical performance, even as it complicates its maintenance.

## Learning from other health professions

This debate is not unique to paramedicine. In anaesthesia, McIntosh and Macario (2008) identified similar tensions, noting that although part-time clinical practice supports retention and well-being, the impact on clinical performance remains uncertain. They questioned whether reduced exposure might limit opportunities to manage rare events or complex procedures – a concern equally relevant to paramedics who might encounter high-acuity cases infrequently.

Other physician-focused studies provide further insight. Kato et al. (2021) found that doctors working fewer clinical days in hospital were associated with higher patient mortality, suggesting that reduced clinical time could influence outcomes. Conversely in primary care, Panattoni et al. (2015) and Kegreiss et al. (2023) showed that while part-time work may reduce continuity and access to care, patient satisfaction often remains stable or even improves. This nuance underscores that competence is multi-faceted: it involves technical ability, decision making and professional judgement, not merely hours logged.

For nurses, the literature highlights both risks and rewards. Jamieson et al. (2008) described part-time nursing as a process of adaptation, where individuals recalibrate their professional identity and capacity; while these findings reflect the situation nearly two decades ago, they provide useful historical context for understanding ongoing professional adaptation. More recent studies, such as Flodgren and Bidonde (2019), have echoed this ambivalence but have also noted that part-time work can positively influence psychological well-being by reducing emotional exhaustion and burnout. Edwards and Robinson (2004) warned that, without structural reform, expanding part-time workforces may lead to underutilisation of skilled professionals. Across professions, there is a consistent theme: clinical exposure matters, but competence is multifaceted, and part-time work can have important benefits for staff well-being. These patterns, observed historically in nursing, may also be relevant to other professions, including paramedicine, as flexible working arrangements become more common. A notable distinction, however, is that many health professions have historically offered protected CPD, supervision or clinical upskilling time within contracted hours, though these provisions have increasingly come under pressure across the wider health sector. Paramedicine has not traditionally benefitted from such structures, leaving clinicians – especially those working part time or across multiple roles – to maintain clinical currency alongside competing professional obligations, often outside paid time. Foundational theorists highlight why this matters: professional legitimacy relies on a continually renewed knowledge base (Freidson, 2001), and competence is dynamic, requiring sustained, supported learning (Eraut, 1994). These perspectives reinforce that structured CPD is not optional but essential, underscoring the need for equitable approaches to maintaining competence within paramedicine.

## Maintaining competence: opportunities, institutional support and self-directed solutions

To help mitigate the challenges faced by dual- and multi-role paramedics, clinical employers and higher education institutions can work together to reduce structural barriers and create flexible pathways. This already exists in the form of lecturer-practitioner and clinical-academic roles within some healthcare organisations. Expanding such arrangements through secondments, joint appointments or formal dual-role pathways can allow paramedics to routinely maintain competency in both clinical and academic domains, supporting smoother transitions between roles and reinforcing professional identity.

Some ambulance services employ minimum shift requirements for paramedics who work less than full time. However, such thresholds lack an empirical basis. Competence depends not only on frequency of exposure but also on the quality of CPD and simulation training (low-frequency, high-acuity events), reflective debriefing, peer feedback and mentorship. This aligns with Ericsson (2004) and Ericsson et al.’s (1993) concept of ‘deliberate practice’: structured, feedback-driven, goal-orientated repetition as the foundation for maintaining expertise. Evidence underpinning the most recent European Resuscitation Council Guidelines indicates there are a broad range of educational interventions that improve competence and, more importantly, reduce patient mortality (Nabecker et al., 2025). For paramedics balancing multiple professional roles, competence might be sustained through CPD rather than sheer clinical volume.

Many universities offer self-managed scholarly or clinical activity, such as allocated days to maintain clinical skills, but these are often underutilised due to lack of awareness or uncertainty about how to apply them alongside academic duties. Universities and NHS organisations share responsibility for enabling this balance through supportive rostering, protected clinical and research time, recognition of dual contributions and encouragement of strategic use of self-managed opportunities, helping paramedic academics sustain competence across clinical and academic roles.

## Conclusion

Paramedicine has evolved into a multi-dimensional profession, with clinicians increasingly working across educational, research and operational settings. Reduced clinical exposure is therefore no longer uncommon and can produce understandable concerns about skill retention, confidence and professional identity. While clinical academics bring enhanced skills – from evidence awareness to specialist insight – they also face practical and structural barriers to maintaining clinical currency. These concerns extend far beyond research and academic paramedics, reaching rural practitioners, those on secondment, those returning from prolonged absence and even full-time clinicians affected by system pressures.

As the profession continues to grow, the priority should not be to question the legitimacy of part-time or portfolio practice but to develop evidence-informed mechanisms that support competence across varied roles. We need to move beyond arbitrary shift quotas towards more nuanced, equitable approaches that include protected CPD time, structured refreshers, clear collaboration between organisations and deliberate practice. This shift in perspective will help paramedics remain confident and competent, regardless of how their careers evolve, and will strengthen the profession’s ability to adapt, innovate and deliver safe, high-quality care.

## Conflict of interest

CW is an Associate Editor for the *British Paramedic Journal*. LP is on the Editorial Board for the *British Paramedic Journal*. DF is an Associate Editor for *Paramedicine*.

## Statement of generative AI in scientific writing

The authors did not use a generative artificial intelligence (AI) tool or service to assist with preparation or editing of this work.
